# From Early Signals to Systemic Decline: Physiological Defense Landscape of *Agave tequilana* in the *Fusarium oxysporum* Pathosystem

**DOI:** 10.3390/plants15020233

**Published:** 2026-01-12

**Authors:** Diego E. Navarro-López, Julio César López-Velázquez, Antonia Gutiérrez-Mora, Mayra Itzcalotzin Montero-Cortés, Martin Eduardo Avila-Miranda, Norma Alejandra Mancilla-Margalli, Elizabeth Sánchez-Jiménez, Miriam Irene Jiménez-Pérez, Jorge L. Mejía-Méndez, Joaquín Alejandro Qui-Zapata

**Affiliations:** 1 Escuela de Ingeniería y Ciencias, Tecnológico de Monterrey, Epigmenio González 500, San Pablo, Santiago de Querétaro 76130, Mexico; diegonl@tec.mx (D.E.N.-L.); mejia.jorge@tec.mx (J.L.M.-M.); 2 Departamento de Ingeniería Química, Industrial y de Alimentos, Universidad Iberoamericana, Ciudad de México, Prolongación Paseo de la Reforma 880, Lomas de Santa Fe, Ciudad de México 01219, Mexico; julio.lopezv@ibero.mx; 3 Biotecnología Vegetal, Centro de Investigación y Asistencia en Tecnología y Diseño del Estado de Jalisco AC, Camino Arenero 1227, El Bajío, Zapopan 45019, Mexico; agutierrez@ciatej.mx (A.G.-M.); elisanjim@yahoo.com.mx (E.S.-J.); 4 Tecnológico Nacional de México/Instituto Tecnológico de Tlajomulco, Km 10 Carretera San Miguel Cuyutlán, Circuito Vicente Fernández Gómez, Tlajomulco de Zúñiga 45640, Mexico; mayra.mc@tlajomulco.tecnm.mx (M.I.M.-C.); martin.am@tlajomulco.tecnm.mx (M.E.A.-M.); norma.mm@tlajomulco.tecnm.mx (N.A.M.-M.); 5 Escuela de Medicina y Ciencias de la Salud, Tecnológico de Monterrey, Av. General Ramon Corona 2514, Zapopan 45201, Mexico; miriamjim@tec.mx

**Keywords:** *Agave tequilana*, *Fusarium oxysporum*, plant defense mechanisms, pathogenesis, agave wilt, tequila

## Abstract

The agave wilt associated with *Fusarium oxysporum* (Fox) is a major disease of blue agave (*Agave tequilana* Weber var. *azul*), used to produce “Tequila” in Mexico. Little is known about the *A. tequilana*-*F. oxysporum* interaction yet understanding defense mechanisms against the pathogen is necessary for control strategies. During early Fox infection, plants trigger defense mechanisms to interrupt the compatible interaction, while Fox’s pathogenesis mechanism interacts with plant response. This study evaluated plant defense mechanisms induced by Fox in *A. tequilana* and their interaction with fungal pathogenesis. For this, an *A. tequilana* pathogenic strain (FPA), and the non-*A. tequilana* pathogenic strains FNPA and FOL were utilized. Early defense mechanisms evaluated were hypersensitive response (HR) and cell wall strengthening in agave roots. Resistance mechanisms evaluated included pathogenesis-related proteins (PR proteins), phytoanticipins and phytoalexins. For early defense, induced HR was greater with FPA than other strains. Cell wall strengthening was found in agave roots, plants responded differentially to different strains. Initial response to FPA and FOL was similar in PR proteins, phytoalexins and phytoanticipins production. However, the response differentiated with FOL over time, indicating an incompatible interaction. The study identified effective and ineffective defense responses of *A. tequilana* to Fox infection, where FPA exhibited compatibility and caused unregulated ROS and PCD, early inhibition of PR activity, extensive lignification, and saponin detoxification. In contrast, this study unveiled incompatible interactions (FNPA and FOL) because of limited colonization, localized HR with suppressed ROS, early and sustained POX activation, significant callose accumulation, moderate lignification, and phenol–saponin dynamics that help in tissue containment and recovery.

## 1. Introduction

Blue agave (*Agave tequilana* Weber var. azul) underpins a highly valuable agro-industry and is increasingly explored for broader bio-industrial uses due to its drought resilience and carbohydrate accumulation [1]. Tequila holds a protected designation of origin (PDO), and Mexican Standard NOM-006-SCFI-2005 recognizes blue agave as its sole authorized raw material. Beyond tequila, blue agave and related species have become priority agro-industrial crops due to their drought resilience, low water demand, and high carbohydrate accumulation. Crucially, because blue agave is not a staple food and typically requires no irrigation, it represents an attractive feedstock for biofuel R&D and other value-added bioproducts [2].

Besides high plant production, wilt caused by *Fusarium oxysporum* (Fox), a challenging soil-borne pathogen, has become a significant obstacle to plant survival and raw material supply [3]. Most research on agave wilt has focused on epidemiological, etiological, and pathogenicity aspects at the field level [4], while the interaction between *F. oxysporum* and *A. tequilana* has been marginally addressed [5,6]. Although Fox is a well-studied soil-borne pathogen, the early physiological defenses and downstream resistance responses of *A. tequilana* remain poorly understood. This knowledge gap limits the development of targeted control strategies and the identification of reliable phenotypic or biochemical markers for resistance, tolerance, and management [7]. Fox can display a series of multifaceted mechanisms that compromise plant health and productivity by inducing wilting and death [8]. For instance, it has been documented that Fox decreases plant health by rapidly colonizing the vascular tissues, disrupting water and nutrient transport when interacting with root systems [9]. When penetrating the xylem and the vascular system, the existence of Fox can also occur in the degradation of cell walls, vascular discoloration, and tissue decay [10], which can be attributed to its capability of modulating hormonal pathways (e.g., abscisic and salicylic acid) involved in defense, stress, plant development responses [11].

From a molecular perspective, it has been noted that, in compatible plant–pathogen interactions, initial cellular barriers and signaling often fail to contain infection, whereas incompatible interactions typically feature a rapid hypersensitive response (HR) with a reactive oxygen species (ROS) burst [12], localized programmed cell death (PCD) events are followed by resistance-associated mechanisms [13,14], including the induction of pathogenesis-related (PR) proteins and accumulation of specific secondary metabolites such as phytoalexins and phytoanticipins that collectively restrict pathogen growth and spread [15]. In agave, the mechanisms induced during interactions between compatible and incompatible *Fusarium* strains remain unresolved.

Models for evaluating the interaction between *Fusarium* strains with plants can be categorized into classical and non-classical. The former category encompasses approaches that emphasize gene-for-gene interactions where specific plant resistance genes are associated with specific pathogen Avirulence genes [16]. Because of this, classical models often present localized responses in affected tissues rather than reflecting a systematic event. Contrary, the latter classification, comprehend models that recognize resistance as a generalized event towards various strains that can also be associated with soil moisture, temperature, and nutrient availability as factors that can predispose the plant to infection [17].

In this study, we used a comparative framework to dissect agave defenses by challenging plants with Fox strains spanning a pathogenicity spectrum: (i) a strain pathogenic to agave (FPA; compatible interaction), (ii) a non-pathogenic strain to agave (FNPA; incompatible interaction), and (iii) a tomato-specific pathogenic strain, *F. oxysporum* f. sp. *lycopersici* (FOL). This evaluation was divided into assessing mechanisms triggered early in the interaction, such as HR, which involves ROS production, and the induction of PCD. It also included cell wall strengthening, assessed by callose and lignin accumulation, and observations of the Fox infection process within the plant. Resistance mechanisms related to Fox, such as the production of PR proteins, the accumulation of phytoanticipins, and the production of phytoalexins, were also evaluated. From these assessments, the *A. tequilana* plant’s response to Fox infection was characterized.

## 2. Results

### 2.1. Early Stage of Infection of Fox and Symptoms Development

**Figure 1:**
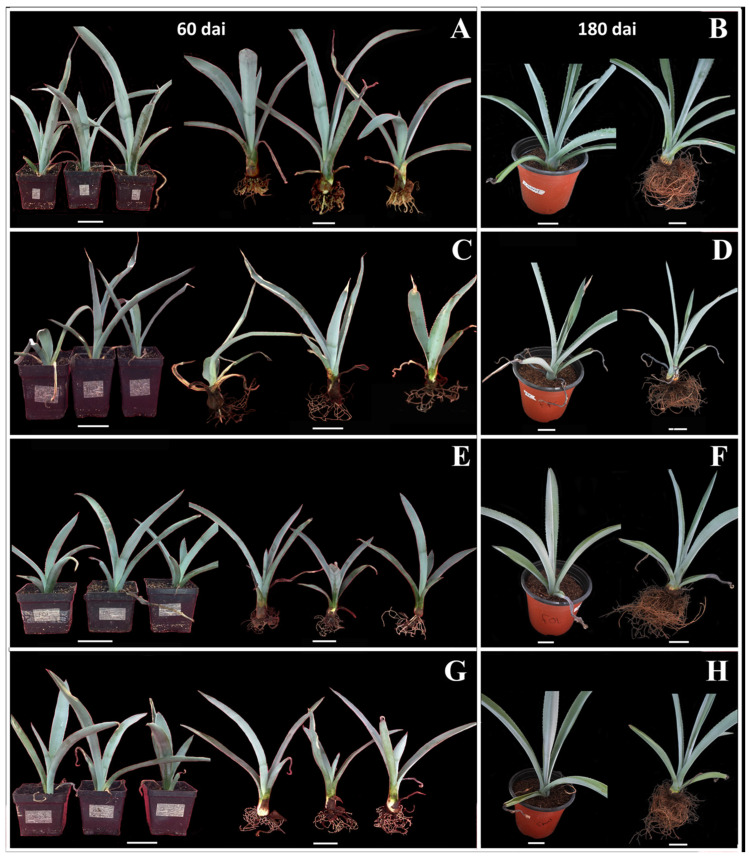
Wilt symptoms in *A. tequilana* after inoculation with Fox. (**A**,**B**), controls (sterile water) at 60- and 180-days after inoculation (dai); (**C**,**D**), pathogenic strain FPA at 60 and 180 dai; (**E**,**F**), tomato-specific strain FOL at 60 and 180 dai; (**G**,**H**), non-pathogenic strain FNPA at 60 and 180 dai. Scale bar: 5 cm.

*A. tequilana* plants challenged with three strains of Fox displayed strain-dependent symptom progressions. At 60 days after inoculation (dai), plants inoculated with the agave-pathogenic strain (FPA) showed clear wilting (see Figure 1C), with apical necrosis, some individuals having completely necrotic leaves, and reduced root volume with pronounced lignification compared to the control (see Figure 1A). At 180 dai, wilting intensified (see Figure 1D): pronounced leaf curl, increased leaf necrosis, and a further reduction in root volume compared to the control (see Figure 1B). In contrast, plants inoculated with the tomato-specific pathogenic strain (FOL) showed mild wilting at 60 dai (Figure 1E)—typically a single necrotic leaf per plant, occasional apical necrosis, and slight curl—with lignified roots and moderate root volume loss compared to the control (see Figure 1A). At 180 dai, wilting was limited or reversed (see Figure 1F), indicating partial recovery. Plants inoculated with the non-agave pathogenic strain (FNPA) did not show significant wilting at 60 dai (see Figure 1G), except for sporadic apical necrosis and no apparent root damage; at 180 dai, they lacked wilting symptoms and recovered from mild early signs (see Figure 1H). Controls remained asymptomatic throughout (see Figure 1A,B).

#### Physiological Response of Plant Defense Induction

**Figure 2:**
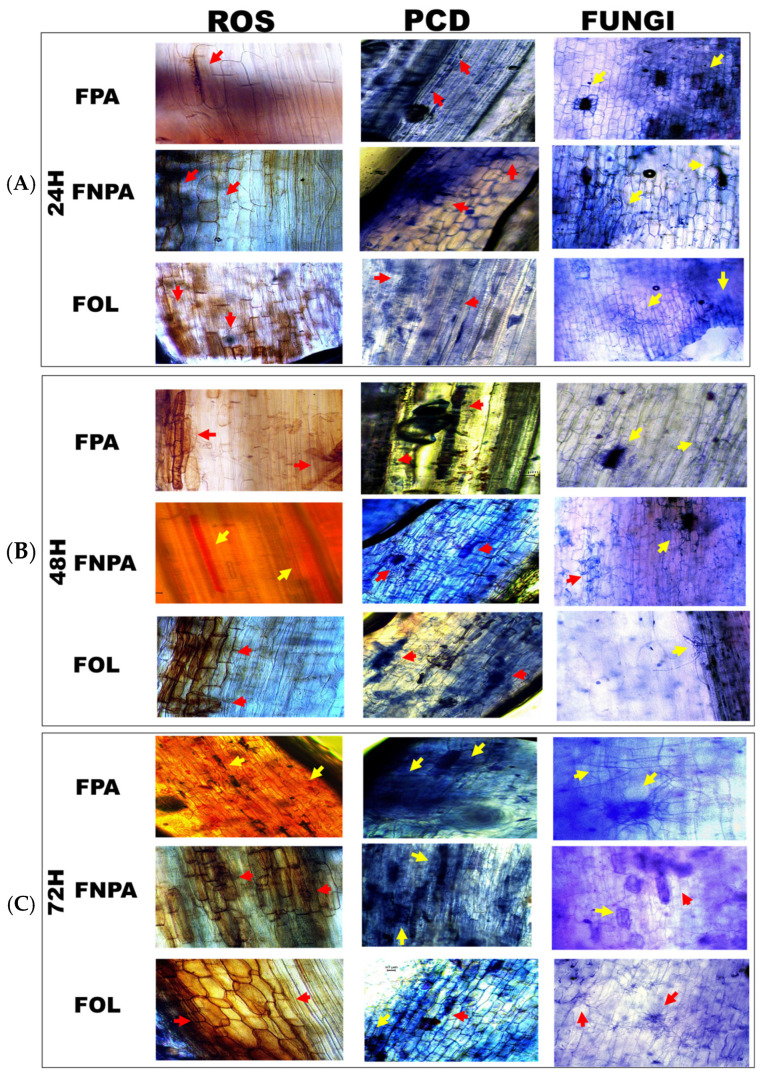
Early responses in *A. tequilana* roots after inoculation with *F. oxysporum*. (**A**) ROS: accumulation of reactive oxygen species; (**B**) PCD: programmed cell death; (**C**) FUNGI: mycelial localization. Strains: FPA, pathogenic to *A. tequilana*; FOL, *F. oxysporum* f. sp. *lycopersici* (specific pathogen of tomato); FNPA, nonpathogenic to *A. tequilana*. Arrows indicate the sites of ROS accumulation, presence of cell death, and fungal mycelium, respectively.

Three early root defense responses were assessed: reactive oxygen species (ROS) accumulation, programmed cell death (PCD), and fungal colonization [18]. ROS (H_2_O_2_) levels were visualized using DAB (Figure 2A). When the agave roots were infected with the FPA strain, the signal was low and focal at 24 h after inoculation (hai), increased at 48 hai, and was high and broadly distributed at 72 hai. In plants inoculated with FNPA, it remained low at 24–48 hai and was moderate but localized at 72 hai. In plants inoculated with FOL, the signal was high from 24 hai, with a wide distribution confined to distinct cell groups, and it increased at 48–72 hai without losing this focal pattern. PCD was determined by trypan blue (Figure 2B). In plants inoculated with FPA, high and extensive PCD was already observed at 24 hai, with further increases at 48 and 72 hai in both distribution and number of affected cells. In plants inoculated with FNPA, PCD was localized at 24–48 hai and increased at 72 hai, although it remained limited and to a lesser extent than in plants inoculated with FPA. In agave roots, infected with the FOL strain, PCD was detected already 24 hai, with a progressive increase at 48–72 hai in cell groups distributed throughout the root.

The presence of hyphae in the root was examined under a microscope (Figure 2C). In agave roots, infected with the FPA strain, extensive colonization with abundant mycelium was observed at 24 hai; by 48–72 hai, the mycelium spread and no longer remained confined to a few cells, covering large areas of the root. In plants inoculated with FNPA, at 24 hai, only a few cells contained hyphae, and extracellular mycelium was predominant. Between 48–72 hai, the mycelium increased but remained mainly outside the cells, with limited colonization (a pattern that differs from plants inoculated with FPA). In agave roots, infected with the FOL strain, the pattern was like plants inoculated with FNPA at 24–48 hai, with abundant extracellular mycelium and few colonized cells. However, at 72 hai, the mycelium became more abundant and spread more widely, although still with few colonized cells, unlike the pattern seen in plants inoculated with FPA.

**Figure 3:**
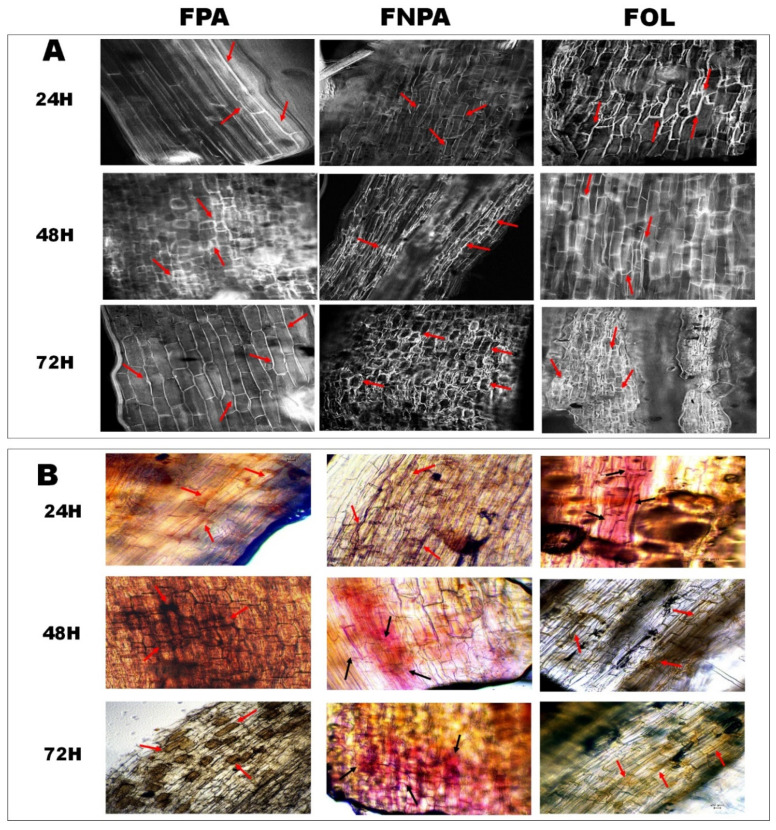
Cell wall strengthening in *A. tequilana* roots after inoculation with Fox. (**A**) callose accumulation; (**B**) lignin production and accumulation. Strains: FPA, pathogenic to *A. tequilana*; FOL, *F. oxysporum* f. sp. *lycopersici* (specific pathogen of tomato); FNPA, nonpathogenic to *A. tequilana*. Arrows indicate sites of callose or lignin accumulation.

Additionally, cell wall strengthening, callose accumulation, and cell wall lignification were assessed (see Figure 3). Callose accumulation was stained with aniline blue and observed under a fluorescence microscope (see Figure 3A). For agave roots infected with the FPA strain, slight callose accumulation was observed in some root cells at 24 hai. At 48 hai, a significant increase in callose accumulation was observed, which continued until 72 hai. For agave roots infected with the FNPA strain, slight callose accumulation was observed at 24 hai, which progressively increased at 48 and 72 hai. This accumulation was lower than in plants inoculated with FPA. In plants inoculated with FOL, significant callose accumulation was observed at 24 hai, which progressively increased at 48 and 72 hai. This treatment showed the highest callose accumulation of all treatments. For cell wall lignification, phloroglucinol staining was performed (see Figure 3B). For agave roots infected with the FPA strain, a slight lignin presence was observed in some root cells at 24 hai; at 48 hai, a considerable increase in lignin accumulation was observed, extending to a greater number of root cells by 72 hai. For agave roots infected with the FNPA strain, lignification was observed in a greater number of root cells at 24 hai, although no increase in lignification was observed at 48 hai. Finally, at 72 hai, a considerable rise in lignification accumulation was observed, although localized to specific sections of the root. For agave roots infected with the FOL strain, at 24 hai, lignification was observed in a few root cells. At 48 hai, an increase in the sites of lignification was observed. Finally, at 72 h, it was observed that these lignified sites showed no significant increase.

### 2.2. Biochemical Consolidation for Defense During A. tequilana-F. oxysporum Interaction

**Figure 4:**
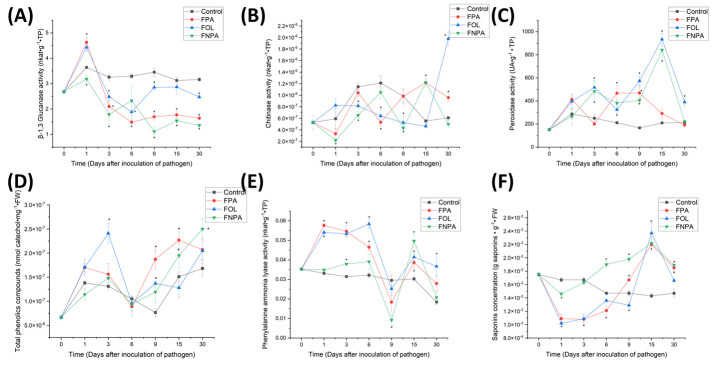
Defense responses in *A. tequilana* roots after inoculation with *F. oxysporum*. (**A**–**C**), PR protein activities: (**A**), chitinases; (**B**), β-1,3-glucanases; (**C**), peroxidases. (**D**–**F**), defense-associated metabolites/enzymes: (**D**), total phenolic compounds; (**E**), phenylalanine ammonia-lyase (PAL); (**F**), saponins (phytoanticipins). Strains: FPA, pathogenic to *A. tequilana*; FOL, *F. oxysporum* f. sp. *lycopersici* (specific pathogen of tomato); FNPA, nonpathogenic to *A. tequilana*. Asterisks (*) indicate statistically significant differences compared to the control (*p* < 0.05).

Late or resistance-related defense mechanisms are local and systemic responses that involve the production of pathogenesis-related proteins (PR proteins), the accumulation of phytoanticipins, and the synthesis of phytoalexins. These mechanisms often rely on an effective early defense response as a prerequisite for their activation [19]. In this study, plant defense mechanisms associated with resistance to *F. oxysporum* were evaluated, focusing on the production of PR proteins, phytoalexins, and phytoanticipins. To assess PR protein production, the activity of β-1,3 glucanases, chitinases, and peroxidases was measured in agave roots (Figure 4). In the evaluation of β-1,3 glucanase activity in agave roots (Figure 4A), it was observed that, in plants inoculated with the FPA strain at 1 day after inoculation with the pathogen (dai), the β-1,3 glucanase activity increased significantly compared to healthy plants. However, by 3 dai, activity decreased significantly relative to the control, and this decline persisted through the subsequent days evaluated up to 30 dai. In plants inoculated with the FNPA strain, it was observed that by 1 dai, β-1,3 glucanase activity decreased relative to the control, and this decrease in activity was maintained from 3 dai to 30 dai. In plants inoculated with the FOL strain treatment, it was observed that by 1 dai, β-1,3 glucanase activity increased significantly compared to the control; by 3 dai, a significant decrease in activity was observed compared to the control, which persisted until 9 dai. Subsequently, at 15 dai, there was no significant difference from the control, and again, a decrease in activity was observed compared to the control at 30 dai. Although the behavior with respect to plants inoculated with FPA is similar, this equality was maintained only until 6 dai; from 9 dai onwards, a significant difference was observed between plants inoculated with FPA and those inoculated with FOL, with β-1,3 glucanase activity higher than that of plants inoculated with FPA and close to the level of control plants.

In the evaluation of chitinase activity in agave roots (Figure 4B), it was observed that for the treatment of the FPA strain, there was no significant difference with the control in the first dai (1 and 3 dai). It was not until 6 dai that a decrease in activity was observed, an increase to the control level at 9 dai, and a significant increase in activity compared to the control, which remained at 15 and 30 dai. In plants inoculated with the FNPA strain, chitinase activity decreased from 1 dai to 3 dai. At 6 dai, activity increased to the control level, and at 9 dai, it decreased again. At 15 dai, activity increased, and at 30 dai, it returned to the control level. In plants inoculated with the FOL strain, chitinase activity decreased from 3 dai onward and continued to decline through 9 dai. From 15 dai, it increased to the control level, and this increase was greater at 30 dai.

In the evaluation of peroxidase activity in agave roots (Figure 4C), it was observed that in plants inoculated with the FPA strain, there was no significant difference with the control in the first dai (1 and 3 dai). Activity increased substantially until 6 dai, then remained constant until 9 dai. From 15 dai to 30 dai, there was no significant difference compared with the control. In plants inoculated with the FNPA strain, there was no significant difference in power at 1 dai; substantial increases in activity occurred up to 3 dai, and this remained the same until 15 dai. It was not until 30 dai that the control activity level returned. In plants inoculated with the FOL strain, peroxidase activity increased from 3 dai onwards, remained elevated until 30 dai, and peaked at 15 dai.

Regarding phytoalexins, total phenolic compound (TPC) production was used as a global indicator of defense-associated metabolites [20]. In plants inoculated with FPA, content of TPC did not differ from the control until 9 dai, when it increased significantly and remained elevated until 15 dai, returning to control levels by 30 dai (Figure 4D). In plants inoculated with FNPA, TPC remained at control levels until 9 dai and increased by 15–30 dai. In plants inoculated with FOL, TPC increased early (3 dai), decreased to control levels by 6 dai, increased again by 9 dai, and normalized by 15–30 dai. In plants inoculated with FPA, PAL activity showed significant increases by 1–6 dai, a drop to control levels by 9 dai, a rebound by 15 dai, and normalized again by 30 dai (Figure 4E). In plants inoculated with FNPA, PAL increased at 3 dai, decreased to or below control levels at 6–9 dai, and rebounded at 15 dai before normalizing at 30 dai. In plants inoculated with FOL, PAL increased at 1–6 dai, fell to control levels at 9 dai, and increased again at 15–30 dai. For the phytoanticipins, in plants inoculated with FPA, saponins decreased significantly at 1–6 dai, then increased at 9–30 dai (Figure 4F). In plants inoculated with FNPA, there was a slight decrease at 1 dai; from 3 dai, they returned to control levels and increased significantly from 6 dai to 30 dai. In plants inoculated with FOL, they decreased at 1–3 dai, normalized at 6 dai, decreased again at 9 dai, and increased significantly at 15–30 dai, with a pattern very similar to that of plants inoculated with FPA.

**Figure 5:**
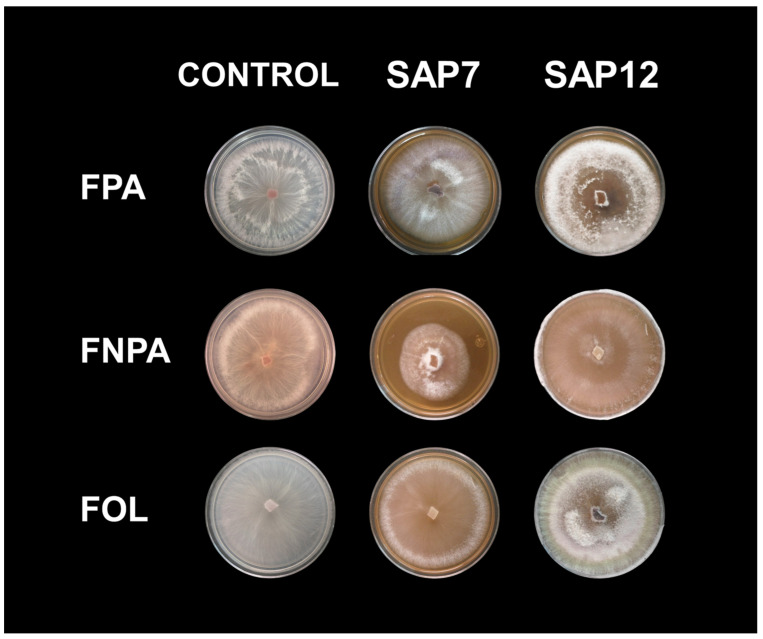
Effect of agave phytoanticipins on the growth of *F. oxysporum*. Control: growth in PDA medium 7 days after inoculation; SAP7: PDA medium supplemented with agave root infusion 7 days after inoculation; SAP12: same medium with agave root infusion 12 days after inoculation. FPA strain, pathogenic to *A. tequilana*; FOL, *F. oxysporum* f. sp. *lycopersici* (specific pathogen of tomato); FNPA, non-pathogenic to *A. tequilana*.

Based on the effects of saponins on the growth of different *Fusarium* strains, a preliminary assessment of their impact on the accumulation of these strains was conducted (Figure 5). It was observed that both FPA and FOL strains grew effectively, without apparent inhibition, in saponin-treated medium, although changes in mycelial morphology were noted. However, the FNPA strain exhibited radial growth defects and showed greater inhibition than the other strains, suggestings a possible pathogenic mechanism that downregulates its colonization in *A. tequilana*.

## 3. Discussion

Agave wilt is one of the main health constraints and a cause of plant death in tequila agave; its detection and control remain a priority [6]. Since *A. tequilana* differs from classic models of interaction with *Fusarium* (multiannual cycle, high drought tolerance, and reversible wilt episodes), understanding Fox pathogenesis and host responses is critical to designing more effective management strategies. The comparative approach employed—contrasting a compatible interaction (FPA) with two incompatible or maladaptive ones (FNPA and FOL)—allowed us to construct a preliminary model that distinguishes effective defenses from inefficient or neutralized responses to the pathogen [21,22].

Here, it was noted that, during colonization, FPA showed rapid progression from 24 h post-inoculation, with abundant mycelium that moved from cellular foci to large root areas, consistent with a compatible interaction and explaining the severe symptoms at 60–180 days. In contrast, FNPA remained mostly extracellular or confined to a few cells for 24–72 h, and although mycelium increased, cell colonization remained limited. FOL advanced intracellularly and extracellularly but at a more restricted rate than FPA, without the exponential growth observed in the agave-pathogenic strain; this was reflected in mild symptoms and a tendency toward recovery at 180 days. The colonization gradient can be attributed to the poor content of virulence factors among FNPA and FOL strains that hamper their growth and capability of evading or suppressing host barriers.

In the early physiological response, the pattern of ROS, PCD, and wall reinforcement was discriminatory. With agave roots, infected with the FPA strain, the ROS signal went from low to very high and diffuse at 72 h, paralleling extensive PCD and increasing colonization, suggesting the absence of an effective HR and, instead, PCD associated with pathogenesis. This outcome is consistent with the action of enzymes and toxins (e.g., FoM35_1 and fumonisin B1) that inhibit defenses, decrease enzymatic activity, or modulate ROS, favoring invasion and pathogenic phenomena [23,24]. With agave roots, infected with the FNPA and FOL strains, ROS accumulation was early but localized; PCD remained focal, and the wall was gradually reinforced. Callose accumulated more intensely in agave roots, infected with the FOL strain, and lignification tended to be moderate and limited. In contrast, in agave roots, infected with the FPA strain, although callose and lignin were present, they were not sufficient to halt fungal advance. POX activation was early and sustained in agave roots, infected with the FOL strain (and sustained in agave roots, infected with the FNPA strain), consistent with ROS containment and functional HR; in agave roots, infected with the FPA strain, POX activation was late and transient, while the fungus likely exploited fungal catalase and peroxidases to detoxify H_2_O_2_, thereby escaping effective HR.

In PR proteins, the profiles of β-1,3-glucanases and chitinases reflected the state of the interaction. In plants inoculated with FPA, a very early increase in β-1,3 glucanases was observed, followed by a sustained decrease, and a delay in chitinases with initial inhibition. This early “shutdown” of PRs is consistent with the action of virulence factors (e.g., FoSSP71) that neutralize defense enzymes and facilitate colonization, while also being associated with dysregulated ROS and extensive PCD [25]. In plants inoculated with FOL, β-1,3 glucanases reached levels close to control levels starting at 9 days, and chitinases recovered late (15–30 days), a pattern consistent with a functional but not exacerbated defense. In plants inoculated with FNPA, both activities were of low overall intensity and fluctuating, suggesting a state of priming rather than a complete response, consistent with their limited colonization. Together with this, the determined low β-1,3-glucanases and chitinases activities can be related to the poor colonization of FNPA and FOL in the roots of *A. tequilana*.

In bioactive compounds, TPC and PAL activity showed different timing patterns across strains. In plants inoculated with FPA, PAL was induced early (1–6 days) and TPC increased at 9–15 days, a behavior that suggests a possible diversion of phenylpropanoid flux toward lignification rather than strict phytoalexins; this aligns with the strong lignification observed and the morphofunctional impact on the root at 180 days. In plants inoculated with FOL, TPC increased early (3 and 9 days), and PAL exhibited a bimodal pattern (1–6 and 15–30 days), suggesting a dynamic and controlled phenylpropanoid activation that can contribute to root containment without chronically compromising root development. In plants inoculated with FNPA strain, late and smaller increases in TPC and PAL were observed, consistent with their low aggressiveness and absence of symptoms. This hypothesis should be validated in future studies with assays that include measurements of lignin precursors or lignin quantification.

In phytoalexins, global TPC measurements indicate defense-associated phenolic reprogramming but do not discriminate against individual molecules. However, the early and transient peaks in plants inoculated with FOL, compared to the delayed peaks in plants inoculated with FPA, support the idea of an earlier and more effective induction of phenolic metabolites in the incompatible interaction. Validation of specific phytoalexins will require targeted analyses (HPLC/LC-MS) to link induced peaks to antifungal bioactivity.

Among phytoanticipins, saponins showed a notable pattern of compatibility. In plants inoculated with FPA and FOL strains, there was an early decline (1–6/9 days) followed by recovery and an increase (15–30 days); in plants inoculated with FNPA strain, the initial decline was slight, recovery occurred from 3–6 days, and the increase was sustained. The early reduction in plants inoculated with FPA and FOL strains, suggests detoxification or use of specific glycosidases by the pathogen, while the late recovery reflects host compensation. The high and sustained maintenance in plants inoculated with FNPA coincides with this strain’s reduced ability to thrive and with poorer radial growth in root-infused media.

Regarding the saponin effect, the growth of FPA (and FOL) in media supplemented with root infusion—rich in saponins—without marked inhibition could indicate that pathogenic or adapted strains possess detoxification mechanisms (e.g., tomatinases or other β-glucosidases) that allow them to tolerate or degrade these compounds [26]. This trait suggests a key component of virulence: the ability to neutralize agave phytoanticipins, with implications for both understanding compatibility and biotechnological applications in fermentation and bioenergy processes, where saponins interfere [27]. This hypothesis should be validated in future studies with trials that include saponin detoxification assessments and identification of fungal enzymes.

**Figure 6:**
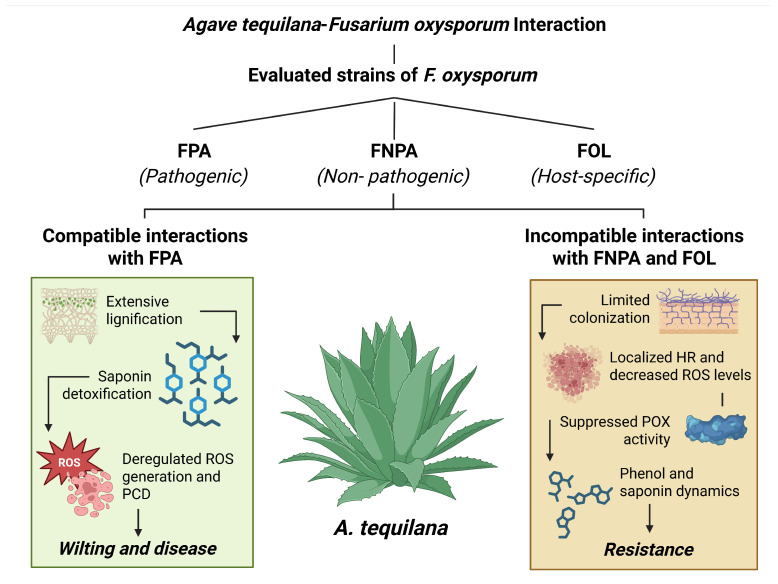
Preliminary diagram proposed for the compatible and incompatible interaction of *Fusarium oxysporum* and *Agave tequilana*. Strains: FPA: pathogenic to *A. tequilana*; FOL: *F. oxysporum* f. sp. *lycopersici* (specific pathogen of tomato); FNPA: nonpathogenic to *A. tequilana*. ROS: accumulation of reactive oxygen species; PCD: programmed cell death; HR: Hypersensitive Response; POX: peroxidase activity.

Taken together, these results allow us to outline a mechanistic scenario: FPA compatibility is associated with rapid colonization, deregulated ROS and PCD, early PR inhibition, enhanced lignification, and saponin detoxification; incompatible interactions (FNPA and FOL) exhibit restricted colonization, focal HR with suppressed ROS, early POX activation, increased callose deposition, and phenol–saponin dynamics conducive to tissue containment. This framework highlights potential intervention points, including genotype selection for effective HRs, management of excessive lignification, and disruption of fungal saponin detoxification mechanisms (Figure 6).

## 4. Materials and Methods

### 4.1. Location of the Study Area

The experiments were conducted at the Unidad de Biotecnología Vegetal and the Laboratorio Nacional PlanTECC of the Centro de Investigación y Asistencia en Tecnología y Diseño del Estado de Jalisco, A.C., Zapopan, Mexico.

### 4.2. Plant Material

In vitro propagation of *Agave tequilana* Weber seedlings (PNov line) was carried out on MS medium supplemented with 2,4-dichlorophenoxyacetic acid (2,4-D, 1 mg/L) and benzyladenine (BA, 10 mg/L). Rooted seedlings were transplanted to a sterile substrate (Peatmoss-Vermiculite, 7:3) and acclimated for two weeks in an incubation room (25 °C, photoperiod 16 h light/8 h dark). Subsequently, they were transferred to a greenhouse in 0.5 L pots and exposed to 10 h of natural light, with irrigation every 3 days and weekly foliar fertilizer applications (Bayfolan® foliar fertilizer, Bayer de México S.A. de C.V., Mexico City, Mexico). Two months after being in greenhouse conditions, they were transplanted again to individual Styrofoam pots with sterile substrate. They were kept under the conditions previously described until their use in the experiments at 4 and 6 months.

### 4.3. Collection of Fungal Strains and Inoculum Preparation

Three Fox strains with different pathogenicity and specificity were used. A *F. oxysporum* strain (FPA), which is pathogenic to *A. tequilana*, was isolated from agave plants with wilting symptoms in Tequila, Jalisco, and whose pathogenicity has been demonstrated in seedlings of *A. tequilana* and *A. cupreata* (“FPC” is the code in the CIATEJ strain collection) [28]. The second inoculum was a strain of *F. oxysporum*, which is non-pathogenic to *A. tequilana* (FNPA). This strain was isolated from an ornamental agave species (*Agave victoria-reginae*) that did not exhibit disease symptoms when inoculated into *A. tequilana* (“FAVR3” is the code in the CIATEJ strain collection) [29]. Finally, a host-specific *F. oxysporum* strain, *F. oxysporum* f. sp. *lycopercisi* (FOL), of tomatoes was included in this work. This strain was provided by Dr. Raúl Rodríguez Guerra from INIFAP-General Terán Experimental Field. All three strains belong to the CIATEJ Plant Biotechnology Fungal Strains Collection. Table 1 presents the utilized strains of *F. oxysporum* in this work.

The fungal inoculum was obtained by culturing each strain on potato dextrose agar (PDA) in a vertical incubator (28 °C, photoperiod of 12 h light/12 h dark). After 15 days of growth, distilled water was added to each Petri dish and the conidia were collected in suspension. These were quantified in a Neubauer chamber and resuspended in sterile distilled water at a concentration of 10^7^ conidia/mL.

### 4.4. Evaluation of Early and Late Induction Defense Responses in A. tequilana by F. oxysporum Infection

The defense mechanisms evaluated were the induction of HR from ROS accumulation, PCD, and cell wall fortification from callose and lignin accumulation. The evaluated defense responses also included monitoring the fungus’s presence in the roots. The seedlings were removed from the pots, the substrate was removed, and they were superficially disinfested with 60% (*v*/*v*) ethanol. Five agave seedlings were directly inoculated by applying a 50 µL drop of the previously described fungal inoculum to marked 1 cm long root sections with no signs of lignification. At least 10 roots from five agave seedlings were inoculated. The inoculated seedlings were placed in a growth room (25 °C, 16-h light/8-h dark photoperiod) in humid chambers. A plant inoculated with a single drop of sterile distilled water served as the control. Root sections were sampled from at least five roots of each seedling inoculated with each *F. oxysporum* strain at 24, 48, and 72 h after inoculation (hai).

#### 4.4.1. Histological Assessment of Fox Colonization in *A. tequilana* Roots

To visualize the presence of the fungus in agave root tissues, roots were placed in a fixative solution (FAA; formalin, acetic acid, ethanol, and distilled water, 10:5:50:35% (*v*/*v*), respectively) and kept at 4 °C. From these samples, root sections were taken and placed in a sodium hydroxide water bath at 50 °C for 30 min. Subsequently, 5% hydrogen peroxide was added and incubated for 30 min. After this time, 0.1N hydrochloric acid was added for 8 min in a water bath, and they were finally stained with 0.5% lactoglycerol-trypan blue (glycerol-lactic acid-distilled water in a 1:1:1 ratio, respectively) for 30 min. At the end of the staining process, lactoglycerol washes were performed to remove excess dye.

#### 4.4.2. Microstructural Dynamics of Defense: ROS, PCD, Callose, and Lignin

##### ROS Production Analysis

For the visualization of ROS in plant tissue, the 3,3’-Diaminobenzidine (DAB) staining was performed following published protocols [30]. Longitudinal sections were cut and transferred to a solution containing 1 mg mL^−1^ DAB (pH 3.5), then incubated in the dark at room temperature for 3 h. The sections were placed on a slide, and hydrogen peroxide (H_2_O_2_) was observed under an Olympus BH2 microscope (Olympus Optical Company, Tokyo, Japan) by the formation of a brown precipitate within the cells.

##### PCD Analysis

The evaluation of PCD was conducted by utilizing trypan blue vital staining. Briefly, agave root samples were cut longitudinally and stained with a 0.5% lactoglycerol-trypan blue solution. They were left to stand for 1 h and were washed immediately afterward with methyl salicylate and water at various dilutions (3:1, 1:1, 1:3) every hour per dilution. The root sections were kept in the stain for 24 h and subsequently washed with lactoglycerol until the excess stain was removed. Finally, observations were made utilizing an Olympus BH2 microscope (Olympus Optical Company, Tokyo, Japan) to validate the presence of dead cells.

##### Callose Accumulation Analysis

The roots were cut longitudinally and placed in 96% ethyl alcohol for 24 h. Subsequently, 1 mL of a 0.5% (*w*/*v*) aniline blue solution dissolved in 0.15 M phosphate buffer was added, and the mixture was incubated for 24 h. Finally, washes were performed with lactoglycerol until excess dye was eliminated. The roots were observed under an epifluorescence microscope (DMRA2; Leica Microsystems, Wetzlar, Germany) using a mercury lamp and a UV/DAPI A2 filter cube (excitation: 370 nm; emission: 455 nm).

##### Lignin Production

To evaluate lignin production, fluoroglucinol/hydrochloric acid staining was performed [31]. Longitudinal sections were made manually and immersed in 1 mL of a 1% (*w*/*v*) phloroglucinol solution in 70% (*v*/*v*) ethanol overnight at room temperature in the dark. The sections were placed on a slide, and a drop of 20% (*w*/*v*) HCl was added to each section, which was then incubated for 5 min. A 1 mL rinse was performed with distilled water, and the samples were visualized under a microscope (Olympus model BH-2, Tokyo, Japan).

#### 4.4.3. Biochemical Profiling of the *A. tequilana* and Fox Interaction

Twenty-four-month-old *A. tequilana* seedlings obtained from in vitro cultures were taken for each treatment and transplanted into individual pots measuring approximately 10 cm^2^ with sterile substrate (peat:vermiculite, 7:3). They were maintained in growth chambers at 30 °C with a photoperiod of 16 h of light and 8 h of darkness. For inoculation, 10 mL of a conidia suspension of *F. oxysporum* was applied directly to the root zone, and the roots were covered with sterile substrate. Control plants received sterile water. Disease severity was graded on a scale of 1 to 5 (1 = healthy; 5 = dead), and root and stem tissue samples were taken at 0, 1, 3, 6, 9, 15, and 30 days after inoculation [5]. For extract preparation, five plants from each treatment were taken and their tissues were frozen in liquid nitrogen, ground to a fine powder, resuspended in 0.1 M sodium phosphate buffer (pH 6.0) at a ratio of 1 g of tissue per 4 mL of buffer, or in methanol, and centrifuged at 8000 rpm for 10 min at 4 °C. The supernatant was used as a crude extract for subsequent enzyme and bioactive compound assays.

##### PR Proteins Analysis

To determine these PR proteins with β-1,3-glucanase activity, the colorimetric method for detecting reducing sugars at 515 nm was used, based on the product of enzymatic hydrolysis and the reduction in DNS (3,5-dinitrosalicylic acid). The quantification was performed using a calibration curve with glucose (0–200 µg/mL), and the activity was reported in nkat per g of fresh weight. 1 nkat is defined as 1 nmol of D-glucose released from laminarin per second, under the assay conditions. This was determined according to published protocols [32], The read was carried out in a spectrophotometer (Thermo Fisher Scientific, Multiskan Go FI-01620, Vantaa, Finland) to 515 nm. For chitinase activity, a colorimetric method was used to measure the formation of N-acetyl D-glucosamine (NAG) produced by the combined hydrolytic activity of chitinases, using colloidal chitin as the substrate. This was performed following published protocols [32]. The NAG concentration was calculated using a standard curve with known concentrations (1–30 mM) of NAG dissolved in sodium phosphate buffer pH 6.0. The chitinolytic activity was expressed in nanokatals per gram of fresh weight. One nkat is defined as 1.0 nmol of NAG produced per second at 37 °C. The read was carried out in a spectrophotometer (Thermo Fisher Scientific, Multiskan Go FI-01620, Vantaa, Finland) to 490 nm.

##### POX Analysis

POX activity was performed using guaiacol as substrate and H_2_O_2_ as co-substrate. A 0.01 mL sample of enzyme extract was mixed with the enzyme extract, and the variation in absorbance per minute was quantified. One unit of peroxidase activity (1 AU) was defined as the change in absorbance of 1 unit per second, expressed per gram of fresh weight. The determination of POX activity was assessed considering published protocols [33]. The read was carried out in a spectrophotometer (Thermo Fisher Scientific, Multiskan Go FI-01620, Vantaa, Finland) to 436 nm.

##### Induced Defense Markers Analysis

For the evaluation of PAL activity, the quantification was the result of non-oxidative deamination of phenylalanine to trans-cinnamate, measured at 290 nm over a period of 3 h. An internal control that did not contain the substrate (L-phenylalanine) was included to correct the reaction for the presence of endogenous substrate. The result was reported in nanomoles of phenylalanine used per hour per gram of fresh weight. The analysis of PAL was executed following published protocols [34], considering a Multiskan GO microplate reader (Thermo Fisher Scientific, Vantaa, Finland) for determining absorbances. For the phytoalexins measurement, the TPC of the methanolic extract was prepared and quantified with Folin–Ciocalteu reagent (Sigma-Aldrich, St. Louis, MO, USA) for 5 min. A 1:5 dilution was prepared with methanol and quantified at 650 and 725 nm. Quantification was performed as published [35], using a standard curve of known catechol concentrations (0.2–1 mM), and the results were expressed as nmol catechol per gram of fresh weight. For the induction of phytoanticipins, saponin quantification was performed as published [36]. Briefly, the methanolic extract was partitioned with petroleum ether, and the polar fraction was retained for acid hydrolysis with HCl. After neutralization to pH 6.0, reducing sugars were determined using 3,5-Dinitrosalicylic acid (DNS) reagent (Sigma-Aldrich, St. Louis, MO, USA). Total saponin concentrations were estimated using *Quillaja saponaria* saponin standard curves (0.5–3 mg mL^−1^), with one series hydrolyzed with HCl and the other without hydrolysis for result normalization.

#### 4.4.4. Effect of Agave Saponins on the Growth of Fox

To evaluate the effect of agave saponins on the growth of *F. oxysporum*, a decoction of *A. tequilana* roots was prepared by boiling 300 g of root segments (seedlings ≥6 months old) in 500 mL of distilled water for 25 min in a water bath; the extract was then diluted to 1 L with distilled water and used to formulate PDA medium (BD Difco, Sparks, MD, USA, 39 g L^−1^). Discs (~1 cm^2^) with mycelium from parent cultures of FPA, FOL, and FNPA were transferred to plates containing PDA–decoction. The plates were incubated for 3 days in the dark at 30 °C and subsequently incubated under a 16 h light/8 h dark photoperiod at 28 °C. The effect was expressed as the percentage of radial growth of the mycelium relative to the control in PDA (considered 100%).

### 4.5. Statistical Analysis

The data obtained from the enzymatic assays, the quantification of phenols and saponins were analyzed by analysis of variance (ANOVA) followed by a Tukey test with a 95% confidence level using RStudio software (version 2023.12.1).

## 5. Conclusions

Taken together, the results from this study confirm that the contrast between *F. oxysporum* strains with different host ranges and levels of pathogenicity supports a preliminary model for understanding agave wilt disease. This model clearly distinguishes between compatible (FPA) and incompatible (FOL, FNPA) interactions. Compatibility is characterized by rapid, systemic, and extensive colonization, involving a multifactorial process associated with widespread ROS accumulation and broad PCD, insufficient cell wall reinforcement, early reduction of β-1,3-glucanases and chitinases, phenylpropanoid pathway shunting toward extensive lignification, and the fungus’s ability to detoxify saponins. In contrast, incompatible interactions mainly exhibit limited colonization, localized HR with concentrated ROS and PCD, early and sustained peroxidase activation, notable callose buildup, moderate lignification, and phenol/PAL activities that support tissue containment and recovery. The behavior of saponins indicates that their defensive effectiveness depends on the host’s accumulation pattern and timing, as well as on the fungal detoxification. This mechanistic framework provides practical criteria for selection and management: promoting focal HR, early POX, and elevated callose; avoiding chronic lignification that compromises the root; and targeting strategies against phytoanticipin detoxification. This lays the groundwork for biomarkers, standardized bioassays, and an integrated strategy for controlling agave wilt by avoiding chronic lignification that compromises root function and implementing strategies to prevent fungal detoxification of saponins (either by enzyme inhibition or by shifting the saponin profile toward less susceptible structures).

## Figures and Tables

**Table 1:** List of *F. oxysporum* strains used in this work.

Code	Name	Pathogenicity
FPA	*F. oxysporum*	Pathogenic strain to agave
FNPA	*F. oxysporum*	Non-pathogenic strain to agave
FOL	*F. oxysporum* f. sp. *lycospersici*	Specific pathogenic strain to tomatoe

## Data Availability

The data presented in this study are available on request from the corresponding author.
